# Clinical Significance of Human Metapneumovirus in Refractory Status Epilepticus and Encephalitis: Case Report and Review of the Literature

**DOI:** 10.1155/2015/131780

**Published:** 2015-11-18

**Authors:** Aysel Vehapoglu, Ozden Turel, Turkan Uygur Sahin, Nurettin Onur Kutlu, Akın Iscan

**Affiliations:** ^1^Department of Pediatrics, Faculty of Medicine, Bezmialem Vakıf University, Adnan Menderes Bulvarı, Fatih, 34093 İstanbul, Turkey; ^2^Department of Pediatrics Infection, Faculty of Medicine, Bezmialem Vakıf University, Adnan Menderes Bulvarı, Fatih, 34093 İstanbul, Turkey; ^3^Department of Pediatric Neurology, Faculty of Medicine, Bezmialem Vakıf University, Adnan Menderes Bulvarı, Fatih, 34093 İstanbul, Turkey; ^4^Department of Pediatric Emergency, Faculty of Medicine, Bezmialem Vakıf University, Adnan Menderes Bulvarı, Fatih, 34093 İstanbul, Turkey

## Abstract

Encephalitis is a complex neurological disease that is associated with significant morbidity and mortality, and the etiology of the disease is often not identified. Human metapneumovirus (hMPV) is a common cause of upper and lower respiratory tract infections in children. Few reports are available showing possible involvement of hMPV in development of neurologic complications. Here, we describe an infant, the youngest case in literature, with refractory status epilepticus and severe encephalitis in whom hMPV was detected in respiratory samples and review diagnostic workup of patient with encephalitis.

## 1. Introduction

Human metapneumovirus (hMPV), a member of the paramyxovirus family (pneumovirinae subfamily), is a respiratory viral pathogen that causes a spectrum of illnesses that range from asymptomatic infection to severe bronchiolitis and pneumonia in children [1]. It was firstly described by van den Hoogen in 2001 as a human pathogen isolated from young children with respiratory tract disease [2]. Mean age at the first infection is 6 to 12 months and nearly all children become seropositive by 5 years of age. Although hMPV can cause respiratory illnesses in all age groups, more severe disease most often occurs in younger patients, prematurely born children, children acquiring nosocomial hMPV infection, and those with severe chronic underlying diseases [3].

Encephalitis is inflammation of the brain parenchyma and manifests by neurologic dysfunction such as altered mental status, personality changes, and motor or sensory deficits. Viruses and autoimmune disorders are the most frequently diagnosed causes of encephalitis, although no etiology is discovered in the majority of cases, despite intensive testing [4].

Refractory status epilepticus (RSE) is a common and life-threatening neurologic emergency. It heralds a prolonged hospitalization and worse prognosis than treatment-responsive status epilepticus [5]. Neurologic symptoms have been described with infections caused by paramyxoviruses such as Hendra, Nipah, mumps, and measles [6]. There is limited knowledge on association of hMPV infection and neurologic manifestations. Here, we describe a child who presented with status epilepticus and was diagnosed with severe encephalitis whose symptoms were possibly associated with hMPV infection. According to our knowledge, it is the case in the literature of the youngest patient with refractory status epilepticus associated with hMPV infection.

## 2. Case Report

A previously healthy 4-month-old male infant presented to emergency department with ongoing seizure activity. The parents explained that the child had 3 days' history of mild rhinorrhea, cough, and fever. He suddenly experienced twitching at right extremity and eye deviation to the right. On admission, he was somnolent, with body temperature of 37°C, respiratory rate of 24/min, and pulse rate of 120/min, rhythmic. The seizure was unresponsive to rectal diazepam and could only be controlled by bolus midazolam and phenytoin infusion. His EEG tracing reflected right frontal sharp wave release consistent with focal seizure (*multifocal epileptiform activity*). He started to have apneic episodes and was transported to the pediatric intensive care unit. He was placed on continuous video-EEG monitoring. Laboratory examinations were normal except for mild metabolic acidosis. Cerebrospinal fluid (CSF) study results showed pleocytosis (leukocytes 100/mm^3^, all lymphocytes), glucose 51 mg/dL, and protein 24 mg/dL. Blood culture and CSF culture were taken and ceftriaxone and acyclovir treatment was initiated. Serum and urine specimens did not identify any underlying inborn metabolic illness. Seizure control could not be achieved with antiepileptic drugs including levetiracetam, phenytoin, and phenobarbital. On midazolam infusion seizures continued and he was intubated and thiopental sodium infusion was started. After 72 h of intubation and mechanical ventilation thiopental sodium was gradually reduced and then stopped.

Imaging studies including chest-XR and cranial magnetic resonance (MR) showed no abnormalities. No bacterial growth was detected in blood, urine, respiratory, and CSF cultures. CSF herpes simplex (HSV types 1 and 2) DNA was negative by polymerase chain reaction (PCR). Investigation of viral pathogens on nasal scrapings by reverse transcription PCR revealed hMPV whereas other viruses including RSV, influenza, parainfluenza, varicella, human herpes virus-6, enterovirus, and echovirus were negative.

He had no seizures during the next days and was discharged on the 25th day of admission. At follow-up after 1 month of discharge, his neurologic exam was normal but the EEG showed pathologic waves. For this reason, we continued to treat him with antiepileptic drugs including levetiracetam and carbamazepine. No seizure was observed during the next 6 months of follow-up.

## 3. Discussion

Since its discovery in 2001, hMPV has been established as a common cause of respiratory tract infections with a worldwide distribution. Indeed, antibodies to hMPV were detected in archived serum samples as early as 1950 [7]. Most of the hMPV diseases are mild or moderate but some may be severe enough to require pediatric intensive care unit (ICU) admission because of acute respiratory failure or encephalitis. Although rare, a broad spectrum of neurologic disorders ranging from seizures to fatal encephalitis/encephalopathy have been reported in association with upper respiratory tract disease due to hMPV (Table 1). Webster et al. described 15- and 18-month-old toddlers with hMPV infection who presented in status epilepticus and went on to develop respiratory failure. Both patients fully recovered and were discharged with no sequelae [8]. Up to date, three fatal cases of encephalitis with possible association of hMPV were reported.

Pathogenesis of development of CNS symptoms with hMPV is unclear. Possible explanations are that hMPV may cause encephalitis by direct invasion to CNS or by postinflammatory mechanism. One of the strongest evidences of direct invasion comes from the study of Schildgen et al. in 2005 [9]. They reported a previously healthy 14-month-old boy with fatal encephalopathy whose autopsy findings revealed hMPV in brain and lung tissues. In 2012, Fernández et al. identified hMPV in cerebrospinal fluid and nasal wash specimens of a 10-year-old girl with acute encephalitis [10]. On MRI, multiple, well-defined white matter lesions on several regions of abnormal cortical signal intensity were detected which suggests an acute demyelinating complication. She developed severe attention and executive deficits. Among 205 pediatric patients referred to California encephalitis project (CEP) between November 2004 and June 2006, hMPV was detected in nasopharyngeal swabs of 5 patients. Upper respiratory infection symptoms were common. Three had seizures and 1 had ataxia. Not all patients had CSF pleocytosis and hMPV was not detected in CSF in any of them [11].

Whether simultaneous detection of hMPV in the respiratory tract of patients with encephalitis proves that the causative agent is hMPV is debatable. Kaida et al. detected hMPV RNA in nasal mucus and tracheal aspirate samples from 1-year-old girl with encephalitis [12]. The virus strain was classified in group A2. The hMPV strain detected by Schildgen et al. was A1 which could suggest a possible link between A strain and the neurological diseases. Among 1474 respiratory specimens taken from children with suspicion of respiratory infections, hMPV was identified in 5.1% [11]. Seizures were reported in 6.3% of them. Asymptomatic colonization with hMPV is rare. Falsey et al. evaluated nasal secretions from adults with and without respiratory illnesses by RT-PCR for RSV and hMPV to determine if rates of detectable RNA were significantly higher among ill subjects compared to controls. hMPV positivity was 3.4% in ill subjects. Of the 158 control subjects, one was RT-PCR positive for RSV and none tested positive for hMPV [13].

The specific etiology remains unknown in 32% to 75% of encephalitis cases even after thorough evaluation. Nevertheless, it is important to identify a specific etiology, if possible, for treatment, prognostic, and public health purposes. Diagnostic workup includes testing of the CSF (e.g., polymerase chain reaction, IgM antibodies) and/or testing of anatomic sites other than the CNS (e.g., stool culture, serology) for viral pathogens. CSF PCR for particular pathogens is helpful when positive, but negative tests do not necessarily exclude the pathogen. In children who have encephalitis with respiratory tract findings, possible infections with Venezuelan equine encephalitis virus, Nipah virus, Hendra virus, influenza virus, adenovirus,* Mycoplasma pneumoniae*,* Coxiella burnetii, Mycobacterium tuberculosis, *and* Histoplasma capsulatum* should be searched. Given the increasing evidence that hMPV may be one of causative agents rather than an incidental finding, we suggest that it should be tested along with other pathogens in children with status epilepticus and encephalitis.

## Figures and Tables

**Table 1:** Children with acute encephalitis associated with human metapneumovirus infection.

Reference	Age/sex	Previous health condition	Seizure	Respiratory infection symptoms	CSF examination	Positive viral PCR
Schildgen et al. (2005) [9]	14 months/M	Healthy	Yes	No	Normal	Brain and lung tissue

Kaida et al. (2006) [12]	1 year/F	NM	NM	NM	NM	Respiratory

Hata et al. (2007) [14]	6 months/F	Healthy	Yes	No	Normal	Respiratory

Arnold et al. (2009) [11]	3 years/F	Healthy	Yes	Yes	13 WBC	Respiratory
	5 years/F	Healthy	Yes	Yes	Normal	Respiratory
	4 years/F	Healthy	No	Yes	227 WBC	Respiratory
	3 years/F	Healthy	No	No	7 WBC	Respiratory
	4 years/M	Healthy	Yes	Yes	NM	Respiratory

Niizuma et al. (2014) [15]	3.5 years/F	Healthy	Yes	No	2 WBC	Respiratory

Webster et al. (2014) [8]	15 months/F	Healthy	Yes	Yes	Normal	Respiratory
	18 months/F	One febrile seizure	Yes	Yes	Normal	Respiratory

Fernández et al. (2012) [10]	10 years/F	Healthy	Yes	No	52 WBC	Respiratory CSF

Index case	4 months	Healthy	Yes	Yes^*∗*^	100 WBC	Respiratory

NM: no mention.

^*∗*^He did not have respiratory tract symptoms during stay in ICU but the parents had observed mild rhinorrhea, cough, and fever 3 days before admission.

## Abbreviations

hMPV: Human metapneumovirus
CSF: Cerebrospinal fluid.
